# Supplementary material for: Treatment Sequencing Strategies in Advanced Neuroendocrine Tumors: A Review
Source: Cancers (Basel). 2022 Oct 26;14(21):5248. doi: 10.3390/cancers14215248 (PMC9656186; doi:10.3390/cancers14215248)
Supplement: Supplementary file 1 [file cancers-14-05248-s001.zip › cancers-1881771-supplementary.pdf]

SUPPLEMENT

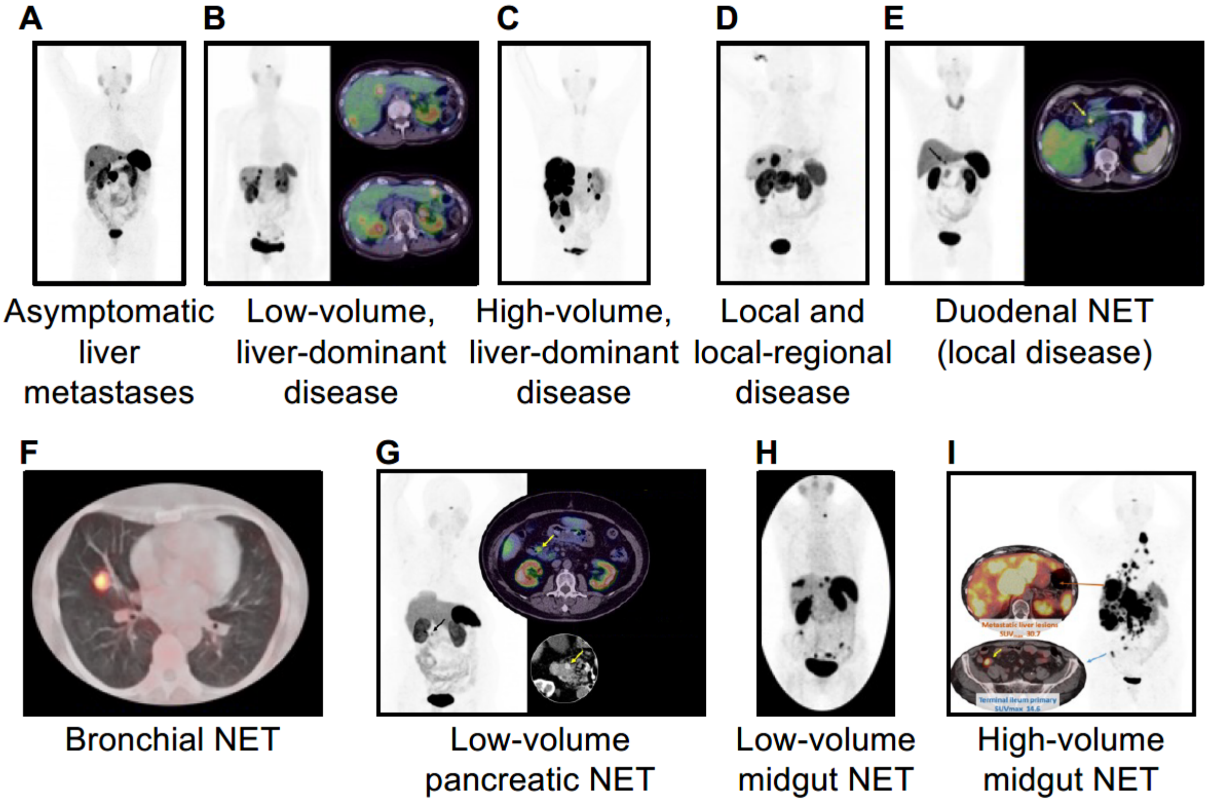

Figure S1. Images of common clinical scenarios encountered in the management of NETs. **A.** ^{68}Ga-DOTATATE PET results (whole-body MIP) in a patient with asymptomatic, low-volume liver metastatic NET. Observation or somatostatin analogs could be considered in this patient. **B.** ^{68}Ga-DOTATATE PET/CT results (left: whole-body MIP; right: cross-sectional CT) in a patient with low-volume, metastatic midgut NET and liver-dominant disease. This patient would be a candidate for loco-regional therapy. **C.** ^{68}Ga-DOTATATE PET results (whole-body MIP) in a patient with high-volume metastatic NET and liver-dominant disease. This patient would be a candidate for PRRT. **D.** ^{68}Ga-DOTATATE PET results (whole-body MIP) in a patient with low-volume metastatic midgut NET with liver-dominant disease. This patient would be a candidate for loco-regional therapy. **E.** ^{68}Ga-DOTATATE PET/CT results (left: whole-body MIP; right: cross-sectional CT) in a patient with early-stage disease and a duodenal primary lesion (indicated by arrows). This patient would be an ideal candidate for surgical resection. **F.** ^{68}Ga-DOTATATE PET/CT results (cross-sectional CT) in a patient with a bronchial NET. This patient would be a candidate for surgical resection of local disease. **G.** ^{68}Ga-DOTATATE PET/CT results (left: whole-body MIP; right: cross-sectional CT) in a patient with a low-volume pancreatic NET (pancreatic head lesion indicated by arrows). **H.** ^{68}Ga-DOTATATE PET results (whole-body MIP) in a patient with low-volume metastatic midgut NET including liver and peritoneal disease. **I.** ^{68}Ga-DOTATATE PET/CT results (left: cross-sectional CT; right: whole-body MIP) in a patient with high-volume metastatic midgut NET and extensive liver metastases. ^{68}Ga, gallium-68; CT, computed tomography; MIP, maximum intensity projection; NET, neuroendocrine tumor; PET, positron emission tomography; PRRT, peptide receptor radionuclide therapy; SUV_{max}, maximum standardized uptake value.
